# Yogurt in Combination with Inactivated *Pediococcus lactis* Modulated Feline Lipid Metabolism, Anti-Inflammation and Fecal Microbiota

**DOI:** 10.3390/ani15111531

**Published:** 2025-05-23

**Authors:** Jintao Sun, Xinshu Gu, Jiaxue Wang, Xiumin Wang, Zhenlong Wang, Hui Tao, Jinquan Wang, Bing Han

**Affiliations:** 1 Key Laboratory of Feed Biotechnology of Ministry of Agriculture and Rural Affairs, Institute of Feed Research, Chinese Academy of Agricultural Sciences, No. 12 Zhong Guan Cun South Street, Haidian District, Beijing 100081, China; 82101235453@caas.cn (J.S.); 19904762097@163.com (J.W.); wangxiumin@caas.cn (X.W.); taohui@caas.cn (H.T.); 2 College of Animal Science and Technology, Jiangxi Agricultural University, No. 1225, Zhimin Avenue, Xinjian District, Nanchang 330045, China

**Keywords:** postbiotic, yogurt, cat, anti-inflammatory, lipid metabolism

## Abstract

Yogurt and postbiotics are both beneficial for intestinal health of pets. This study was conducted to investigate the effects of yogurt with postbiotics in cats. A total of 18 adult cats were randomly divided into three groups, CK group (Control *n* = 6, free feeding and drinking without any probiotic products), Y group (Yogurt, *n* = 6, 50 g/day), and YP group (Yogurt + 2% postbiotics, 50 g/day), with the same environment and diet. On the 21st day, blood and fecal samples were collected and tested for biochemical indices, immunological parameters, inflammatory factors, and fecal flora, respectively. The results showed that feeding postbiotic yogurt to cats significantly reduced the levels of total bilirubin (TBIL), total bile acids (TBA), triglycerides (TG), glucose (GLU), urea nitrogen (BUN), creatinine (CREA), tumor necrosis factor-alpha (TNF-α), and Interleukin-6 (IL-6) (*p* < 0.05), and significantly increased Immunoglobulin A (sIgA) (*p* < 0.05) compared to the CK group. The abundance of *Bifidobacterium* in YP group was elevated, the Shannon violin plots showed that the intestinal flora of the YP group was less dispersed. Notably *Enterococcaceae* and *Enterococcus* were significantly elevated in the Y group (*p* < 0.05) and *Streptococcus salivarius* subsp. *thermophilus* in the YP group (*p* < 0.05). Therefore, this study suggests that yogurt with postbiotic is a good choice for improving intestinal health and immune function in cats and possesses good research and application prospects.

## Simple Summary

At present, there is very little research on yogurt and postbiotics on cats, and yogurt has many benefits for intestinal health in humans, so we want to use feline-derived lactic acid bacteria and postbiotic pairs to ferment into yogurt to explore the benefits for cats.

## 1. Introduction

Fermented milk, as a live probiotic carrier [1], can not only increase the number of probiotics in the intestinal tract [2] and balance the intestinal flora in animals [3], but can also provide a variety of nutrition, like high-quality whey and casein proteins, B vitamins [4], and short-chain fatty acids (butyric and lactic acids) produced during fermentation process, which contribute to gut health [5]. Fermentation of either cow’s milk can serve to regulate lipid metabolism, reduce inflammation, enhance immunity, lower cholesterol, and regulate intestinal flora [6,7]. Although raw cow’s milk can provide high-quality protein for dogs and cats, most dogs and cats are lactose intolerant [8], and diarrhea and abdominal pain will occur after consuming raw cow’s milk [9].

This study investigates the beneficial effects of yogurt on feline gut microbial communities through comprehensive analysis of microbial diversity and abundance. After fermentation by lactic acid bacteria, it can reduce lactose content [10] and reduce lactose intolerance in dogs and cats. Yogurt can improve metabolic health and reduce visceral fat, which has a very good application prospect for obese dogs and cats [11]. The gut microbiota is closely associated with host health, playing critical roles not only in nutrient metabolism, immune regulation, and pathogen defense, but also being implicated in various health conditions including obesity, diabetes, and inflammatory bowel disease (IBD) [12].

Dogs and cats often suffer from loss of appetite and poor food acceptance during the weaning and food change periods, which can lead to diseases such as stunting and growth deformities [13], and could theoretically contribute to behavioral changes in some individuals [14]. Fermented milk is probably the right product to be used as a supplement during weaning and food changes to reduce stress in pets [15]. Yogurt has recently been studied in weaned piglets as a functional supplement to support growth, gut health, and immunity [16].

Postbiotics are non-viable bacterial products or metabolic byproducts derived from the fermentation process of probiotics [17]. Studies on postbiotics have demonstrated their ability to lower plasma cholesterol, triglycerides, and LDL levels in broiler chickens [18] while also promoting beneficial regulation of gut microbiota and enhancing short-chain fatty acid production in dogs experiencing soft stool conditions [19]. Currently postbiotic and fermented milks have been studied in supporting infant growth, boosting infant immunity, regulating recognized intestinal flora, and improving constipation symptoms and emotional state [20,21]. There are fewer studies on feeding cat-derived lactobacilli to cats directly after fermentation of raw cow’s milk and fermented milk with added postbiotics. Given the increasing interest in functional foods for companion animals, particularly for the management of nutritional disorders and immune health, investigating the combined effects of fermented milk and postbiotics particularly using feline-derived bacterial strains, represents a novel approach that may offer species–specific benefits. This study is designed to evaluate the effects of fermented lactobacilli on cats’ health, as well as the synergistic effects of postbiotics with yogurt fermentation.

## 2. Materials and Methods

### 2.1. Experimental Design and Sample Collection

The animal experiment was implemented according to the Animal Care and Use Committee of the Institute of Feed Research of the Chinese Academy of Agricultural Sciences (CAAS) and was approved by the Laboratory Animal Ethical Committee and its inspection of the Institute of Feed Research of CAAS (IFR-CAAS-20250307).

**Figure 1:**
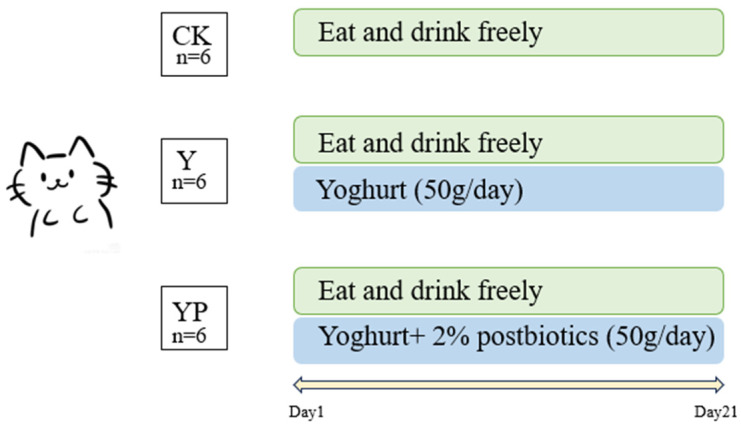
Experimental design.

A total of 18 healthy adult cats of similar weight and age (all around 3 years old, half male and half female, breeds covering Ragdoll, Russian Blue, and British Shorthair, intact, 3.25 ± 0.59 kg) were randomly divided into three groups. In these 21 days, cats were fed with complete cat food (Yantai China Pet Foods Co., Ltd., Yantai, China) once a day in the morning, water added and feces cleaned everyday while interacting with humans. CK group (Yogurt, *n* = 6, 50 g/day), YP group (yogurt + 2% postbiotics, 50 g/day). The test experiment was conducted for 21 days with daily feeding, and the average daily consumption was >40 g in both Y and YP groups (as a complementary diet). The cats were housed in 12 stainless steel cages (160 × 70 × 190 cm) in rooms with a temperature of approximately 23 ± 2 °C. Each room was equipped with adequate food and water, identical litter boxes, and all cats had free access to food and water. On day 21, fresh feces were collected for microbiota analysis and transferred to −80 °C for storage. Blood samples were taken for analysis on day 21 (Figure 1).

### 2.2. Yogurt Fermentation

In our previous study, three strains, *Lactobacillus plantarum* L-27-2, *Pediococcus lactis* L-14-1, and *Enterococcus faecalis* F203, were isolated from healthy cats [22]. *Streptococcus thermophilus* K8 was preserved in our lab. The optimal fermentation ratio of *Streptococcus thermophilus*, *Lactobacillus plantarum*, *Enterococcus faecalis,* and *Pediococcus lactis* was determined to be 5:2:2:1. The fresh fermentation broth of the strains (K8 16 mL/L, L-27-2 6.4 mL/L, F203 6.4 mL/L, and 141 3.2 mL/L) were inoculated into pasteurized raw cow’s milk with the addition of skim milk powder (10 g/L), dextrose (4 g/L) and glucose (4 g/L). *Pediococcus lactis* L-14-1 was incubated at 37 °C for 8–10 h, and the termination acidity was pH ≤ 4.3. *Pediococcus lactis* L-14-1 (10^11^ CFU/g) was inactivated at 80 °C for 20 min.

### 2.3. Blood Biochemical Parameters

In this experiment, about 1–2 mL blood samples were collected from the cats’ saphenous vein by venipuncture on day 21. After the blood samples were allowed to rest for 30 min, the serum was separated by centrifugation at 5000 rpm for 15 min, and was subsequently dispensed. An amount of 100 μL of serum was tested for blood biochemical parameters, using a biochemical analyzer (MNCHIP, Tianjin, China), including total bilirubin (TBIL), creatine kinase, total bile acids (TBA), triglycerides (TG), glucose (GLU), urea nitrogen (BUN), creatinine (CREA), and cholesterol (TCHO).

### 2.4. Analysis of Blood Immunological Parameters and Cellular Inflammatory Factors

The kit assay (Jiangsu Meimian Industrial Co., Ltd., Yancheng, China) was used to test the secretory sIgA, TNF-α, and IL-6 of the blood on day 21 by the ELISA method (OD = 450 nm).

### 2.5. Fecal DNA Extraction

Total DNA was extracted from the fecal samples on day 21, and the concentration of the DNA samples was measured using the E.Z.N.A Mag-Bind Soil DNA kit (Omega, M5635-02, San Antonio, TX, USA) and the Quibit dsDNA HS kit (Thermo, Waltham, MA, USA). The extracted DNA samples were stored at −80 °C for further PCR amplification [9].

### 2.6. PCR Amplification of Amplicons

The PCR products were examined by electrophoresis. 16S rDNA V3-V4 was amplified and sequenced by second generation sequencing at Sangon Biotech Co (Shanghai, China).

The forward primer sequence was CCTACGGGGGNGGCWGCAG, and the reverse primer was GACTACHVGGGGTATCTAATCC. PCR assays were amplified twice. The PCR reaction conditions were as follows: 94 °C, 3 min, 5 cycles at 94 °C, 30 s, 45 °C 20 s, 65 °C 30 s; 20 cycles at 94 °C 20 s, 55 °C 20 s, 72 °C, 30 s; 72 °C 5 min, as referred to methods [9].

### 2.7. Statistical Analysis

The alpha diversity index was determined based on the Shannon index of fecal microbiota. The alpha diversity index is calculated using Mothur (version 1.43.0). beta diversity to assess differences in microbiomes between samples and was often combined with dimensionality reduction methods such as principal coordinate analysis (PCoA) to obtain a visual representation. Differential comparisons are performed using STAMP (version 2.1.3) and LefSe (version 1.1.0) software to identify features that differ significantly in abundance between groups. Correlation coefficients and *p*-values between communities were calculated using Spar CC (version 1.1.0). Evolutionary trees were constructed using Mega (version 7.0.26).

Blood parameters and fecal biomarkers were analyzed using one-way ANOVA followed by Tukey’s post hoc test for multiple comparisons. Data are presented as bar graphs (mean ± standard error), with statistical significance defined as “*p* < 0.05”. All statistical analyses were conducted using IBM SPSS Statistics 25.0 (IBM Corp., Armonk, NY, USA).

## 3. Results

### 3.1. Blood Biochemical Parameters Results

**Figure 2:**
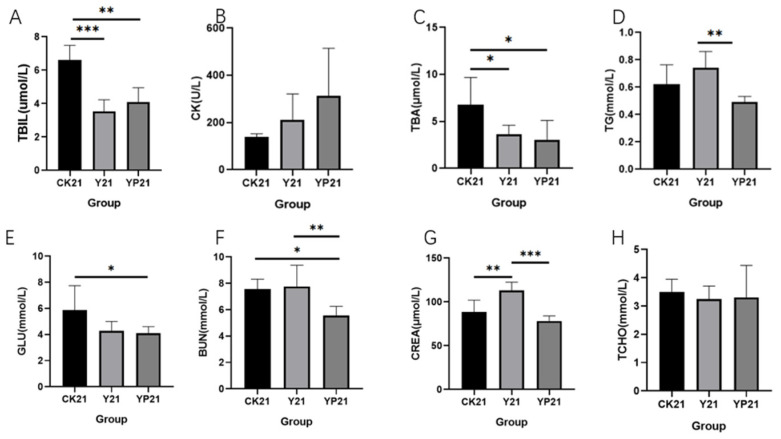
The effects of different groups on the blood biochemical indices. * means *p* < 0.05; ** means *p* < 0.01; *** means *p* < 0.001. (**A**) The serum concentration of TBIL of different treatments; (**B**) the serum concentration of CK of different treatments; (**C**) the concentration of TBA in serum; (**D**) the concentration of TG in serum; (**E**) the serum concentration of GLU of different treatments; (**F**) the serum concentration of BUN of different treatments; (**G**) the concentration of CREA in serum; (**H**) the concentration of TCHO in serum.

The serum levels of TBIL and TBA were significantly reduced in group Y and YP compared with group CK (*p* < 0.05), reducing by 46% and 38%, respectively (Figure 2). There was no significant difference in TCHO levels between the CK group and the treatment groups. There was no significant difference between TCHO and CK, but there was a tendency to increase in CK.

### 3.2. Blood Immune Parameters and Cytokine Results

**Figure 3:**
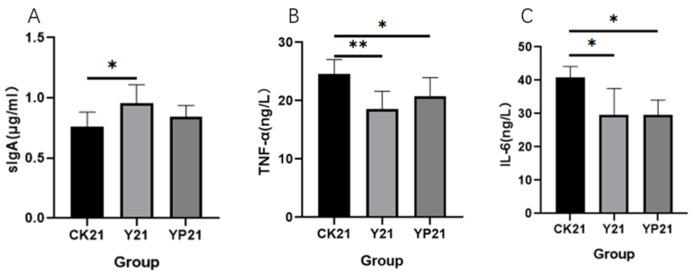
The effects of different groups on immune factors. * means *p* < 0.05; ** means *p* < 0.01 (**A**) The serum concentration of slgA of different treatments; (**B**) the serum concentration of TNF-α of different treatments; (**C**) the concentration of IL-6 in serum.

The results showed that the sIgA level of group Y was significantly increased compared with group CK (*p* < 0.05), rising by 25% (Figure 3). Both the serum levels of TNF-α and IL-6 levels on day 21 in Group Y and group YP were significantly decreased compared with group CK (*p* < 0.05).

### 3.3. Fecal Microbiota Analysis

**Figure 4:**
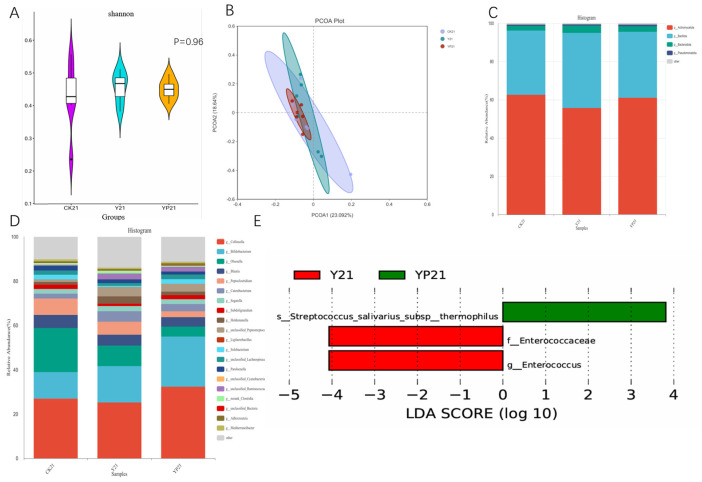
(**A**) Shannon’s indices of the microbial community; (**B**) principal coordinate analysis (PCoA) of the genus level; (**C**) shows the abundance of the phylum level of changes in the fecal microbiota; (**D**) shows the genus level of the changes in the fecal microbiota; (**E**) shows the genus LDA score of the two treatments.

There was no significant difference for α the diversity of microbial community among the three groups (*p* > 0.05), but the Shannon violin plots showed that the intestinal flora of the YP group was less dispersed, which was consistent with the results of the β-diversity of the YP group, and it indicates that the consumption of the probiotic yogurt could lead to the aggregation of intestinal microorganisms (Figure 4). On the phylum level, *p_Bacillota* was elevated in the Y and YP groups relative to the CK group without significance. On the genus level, group Y and YP showed an increased abundance in probiotic Bifidobacterium compared to group CK. Notably *Enterococcaceae* and *Enterococcus* were significantly elevated in the Y group (*p* < 0.05) and *Streptococcus salivarius* subsp. thermophilus in the YP group (*p* < 0.05).

## 4. Discussion

Studies have shown that yogurt could reduce inflammation, enhance immunity, and exert a stabilizing effect on the intestinal flora, which positively affected the health of cats. None of the cats observed any adverse effects or clinical symptoms during the 21-day experiment. In addition to reducing inflammation, the addition of postbiotics had a more prominent effect on lipid metabolism and regulation of gut microbial homeostasis. This was consistent with the anti-obesity results of inactivated *Lactobacillus amyloliquefaciens (CP1563)* [23], *Bifidobacterium animalis lactis* subsp. *lactis* (CECT 8145) [24], and *Laminoplasma pentosum* (LP28) [25] administered orally as postbiotics in human clinical trials [26].

Lipid metabolism and intestinal inflammation in cats is an increasingly common problem, and too much body fat produces insulin resistance, which can lead to serious complications such as diabetes and hepatic lipidosis [27]. TBIL, TBA, TG, GLU, BUN, and CREA are serum biochemicals reflecting body inflammation and lipid metabolism function. Reducing TBIL and TBA can reduce the incidence of hepatitis and cholangitis by adjusting bile acid metabolism [28]. Reducing TG is especially important for hypertriglyceridemic acute pancreatitis [29]. Hyperglycemia is closely related to inflammation. Persistent hyperglycemia promotes the formation of advanced glycosylation end products, increases the release of fibroblast-like synoviocytes, and forms chronic inflammation; regulation of lipid metabolism is closely related [30]. Within the range of healthy values, yogurt significantly reduced TBIL and TBA levels (*p* < 0.05), which suggested that yogurt may be able to improve the effects of bile acid metabolism [22,31], reduce inflammation in the body, improve obesity, and reduce the probability of acute hepatitis [28,32]. The addition of postbiotics was able to reduce significant TC, BUN, GLU, and CREA levels, suggesting that inactivation of *Pediococcus lactis* L-14-1 could alleviate obesity, enhance lipid metabolism, alleviate endocrine disorders, and reduce the probability of pancreatitis, which was in line with our previous study on *Pediococcus lactis* L-14-1 [22], and was also consistent with the effect of postbiotics on metabolic disorders as summarized [33].

SIgA is the most abundant immunoglobulin in the intestinal mucosa and is mainly supplied by milk and intestinal flora [34]. It was found that slgA in milk, can improve the sIgA bacterial group in the intestinal tract, and play a positive promoting role [35]; it was inferred that sIgA in group Y originated from the direct provision of fermented milk.

TNF-α and IL-6 could be the response to the intestinal barrier damage, intestinal inflammation, and the most direct measure [36]. Compared with the CK group, the Y and YP groups could significantly reduce the levels of TNF-α and IL-6 in cat serum (*p* < 0.05), indicating that fermented milk could inhibit the release of pro-inflammatory cytokines, effectively alleviate the inflammatory response of the organism, and promote intestinal health, and this result was consistent with the results of the study of alleviating intestinal inflammation in piglets by feeding piglets with fermented milk [16]. The protective mechanism of yogurt may be to enhance intestinal barrier dysfunction by increasing tight junctions. Yogurt can increase the mRNA levels of claudin-1, ZO-1 and occludin in Caco-2 injury model, and improve the intestinal barrier function damaged by inflammation [37].

Since the main probiotics and postbiotics of fermented milk were initially screened from cat feces, the α-diversity and β-diversity of the cat’s gut microbiota did not change much, but from the point of view of the Shannon index and the β-diversity at the genus level, the YP group obviously brought the gut flora of different individuals within the same group to be more aggregated, and was able to reduce the variability of the gut flora of different individuals. This result was similar to that of the study made on brewer’s yeast postbiotic [38]. This suggests that incorporating postbiotics into yogurt could stimulate the growth of intestinal probiotics, accelerating their proliferation to promote a balanced gut microbiota and enhance its beneficial effects on intestinal health.

On the phylum level, *Bacillota* was improved in Y and YP groups relative to CK group without significance. On the genus level, the Y and YP groups showed an increase in the abundance of the Bifidobacterium compared to the CK group, and Bifidobacterium was showed to enhance the host’s immunity, and anti-inflammatory ability [39], for it could produce SCFAs and other metabolites, which might be beneficial for intestinal mucosa. Increased *bifidobacterial* abundance can manipulate lipid synthesis genes and phosphorylated proteins by regulating signaling pathways such as AMPK/Nrf2, LPS-TLR4-NF-κB, AMPKα/PGC-1α, SREBP-1/FAS, and SREBP-1/ACC to reduce hepatic lipid accumulation and oxidative stress [40]. The increase in *bifidobacteria* coincided with a decrease in lipid markers (TBA, TG, GLU, BUN) and inflammatory markers (TBIL, CREA, TNF-α, IL-6). It was noteworthy that *Enterococcus* were also significantly elevated in the Y group and *Streptococcus salivarius* subsp. *thermophilus* in the YP group, the latter Strain of fermentation F203 was a kind of symbiotic bacteria and produces some antibacterial substances.

In summary, probiotic yogurt with postbiotics could be used as a functional food to prevent obesity or inflammation in domestic cats. However, due to time and animal constraints, the direct addition of postbiotics to cat food was not tested, nor was there a clear distinction made between the direct effects of postbiotics and the effects of interactions between postbiotics and yogurt, which needs to be further investigated in future long-term trials.

## 5. Conclusions

The study showed that the consumption of postbiotic-added yogurt in cats was able to reduce the levels of TBIL, TBA, TG, GLU, BUN, CREA, TNF-α, and IL-6 (*p* < 0.05), demonstrating that postbiotics can synergize with yogurt to reduce inflammation, enhance lipid metabolism, and promote intestinal health in cats. It can improve the immunity of cats by significantly increasing the serum level of sIgA (*p* < 0.05). It also improves gut microbial stability by increasing probiotic abundance and allowing colonization by yogurt-derived strains, which may contribute to long-term gastrointestinal health. Yogurt, being cost-effective, readily available, and nutritionally dense, holds significant potential as an emergency dietary supplement to support feline health, particularly for kittens requiring rapid nutritional intervention. It was demonstrated that postbiotic yogurt has a strong application potential for cats. Meanwhile, further research is needed to evaluate long-term safety, optimal dosage, and effects in larger cat populations.

## Data Availability

The original contributions presented in this study are included in the article. Further inquiries can be directed to the corresponding author.

## Abbreviations

The following abbreviations are used in this manuscript:

MDPI	Multidisciplinary Digital Publishing Institute
DOAJ	Directory of open access journals
TLA	Three letter acronym
LD	Linear dichroism
